# Visibility in Endourologic Workup (VIEW): Comparison of Gas and Saline Cystoscopy

**DOI:** 10.7759/cureus.102207

**Published:** 2026-01-24

**Authors:** Jeison Caruso, Lukas John Hefermehl, Jan Birzele, Uwe Bieri

**Affiliations:** 1 Department of Surgery, Division of Urology, Kantonsspital Baden, Baden, CHE; 2 Department of Urology, Kantonsspital Graubünden, Chur, CHE; 3 Department of Urology, University Hospital of Zurich, Zurich, CHE

**Keywords:** bladder cancer diagnosis, cystoscopic technique, endoscopic visibility, hematuria, porcine bladder model, randomized crossover study

## Abstract

Introduction

Macrohaematuria often compromises cystoscopic visibility. We therefore compared gas-based versus saline cystoscopy in a standardized ex vivo macrohematuria model to assess visibility, procedure completion, and observed procedural performance.

Methods

In a monocentric, two-arm, open-label, randomized crossover pilot, physicians performed cystoscopy on ex vivo porcine bladders modified to mimic human access. Macrohematuria was simulated using a blood-analogue solution. Each bladder was distended with ambient air (180 ml; manual syringes; no intravesical pressure control) or saline (180 ml) in randomized sequence. Three predefined intravesical markings served as visibility targets. The primary endpoint was time to identify all the markings; secondary endpoints included subjective visibility (five-point Likert), perceived visual impairment, ease of use, and iatrogenic trauma (modified Objective Structured Assessment of Technical Skills (OSATS)). Paired and nonparametric tests were prespecified for ordinal outcomes.

Results

Thirteen physicians (urologists and surgical residents) completed both modalities. All participants identified all targets in both conditions. Visibility ratings favored GASCYS (good/excellent 92.3% vs 38.5%; p=0.0019). Ease-of-use ratings uniformly favored GASCYS (p= 0.0012). Perceived visual impairment was lower with GASCYS (trend; p=0.067).

Conclusion

Ambient-air cystoscopy was feasible in a standardized ex vivo macrohematuria model and was associated with improved perceived visibility and usability, highlighting its potential for further investigation in clinical studies. Clinical evaluation needs to define effectiveness, safety parameters (including gas type and pressure control), and indications for gas distension in diagnostic cystoscopy during hematuria.

## Introduction

Background and rationale

Macrohematuria, defined as the presence of visible blood in the urine, is a common and often alarming clinical presentation in urology, frequently prompting urgent evaluation and management [1,2].

The differential diagnosis is broad, encompassing malignancies such as urothelial carcinoma, prostate cancer, and renal cell carcinoma, as well as traumatic injuries, infections, and benign causes, including prostate hyperplasia or urethral strictures [2-9].

Diagnostic cystoscopy remains the gold standard for identifying the source of bleeding within the lower urinary tract [1,2]. However, blood and clots within the bladder can significantly compromise endoscopic visibility during liquid-based cystoscopy [10,11] as ongoing bleeding readily admixes with the distension medium, producing turbidity and optical scattering; consequently, repeated irrigation and clot evacuation are often required and may still yield a non-diagnostic or deferred examination. In clinical practice, continuous bladder irrigation (CBI) is often used as an initial conservative measure to prevent urinary obstruction caused by clot retention [12]. While effective in some cases, CBI can delay definitive diagnosis and treatment, prolong hospital stay, or necessitate rescheduling diagnostic procedures.

An alternative approach is gas-based cystoscopy (GASCYS), which employs gas rather than fluid as the distension medium. First described in the 19th century [13], this technique has been largely supplanted by liquid-based cystoscopy (LICYS) due to advancements in endoscopic instrumentation and imaging. Importantly, this shift was also driven by workflow and technical advantages of fluid distension, particularly the ability to perform continuous irrigation to flush debris and blood, maintain a stable optical field, and support routine instrumentation, whereas gas-based cystoscopy offered limited incremental benefit in typical (non-hematuric) settings.

Nevertheless, the physical properties of gas, namely its lack of miscibility with blood, may confer advantages in visual clarity during hematuria. Despite the theoretical benefit and routine use of insufflation in laparoscopic surgery, the application of gas as a distension medium in cystoscopy has been scarcely investigated, with published data remaining limited to a few case reports and small series [14-17].

Only Ciudin et al. [15] have systematically explored GASCYS under hematuric conditions in a standardized setting. However, the interpretability of these observations is limited because key determinants of visibility (e.g., bleeding load/clot burden and potential sequence effects related to prior irrigation or clearance) were not experimentally controlled, such that a wash-out contribution to the perceived visibility advantage cannot be excluded, as highlighted in subsequent correspondence [18].

No randomized or comparative preclinical data are available to evaluate its feasibility, safety, and diagnostic utility in this context. Accordingly, this preclinical randomized crossover pilot study in an ex vivo porcine bladder model simulating gross hematuria compared GASCYS with LICYS, with the primary objective of measuring the time required to identify predefined intravesical targets and secondary objectives of assessing endoscopic visibility, perceived visual impairment, procedural difficulty/usability, and iatrogenic trauma.

Study objective

Before launching a randomized controlled trial (RCT) in clinical settings, the current study was designed as a preclinical pilot investigation using an ex vivo porcine bladder model. The porcine bladder closely approximates the human bladder in terms of anatomy and compliance, making it a suitable model for endoscopic training and translational research.

This study aimed to compare GASCYS with conventional LICYS under simulated gross hematuria conditions. Specifically, we evaluated objective parameters such as visibility, procedure duration, and iatrogenic trauma alongside subjective assessments by participating clinicians. Based on the distinct physical behavior of gas versus fluid in the presence of blood, we hypothesized that GASCYS would result in superior endoscopic visibility without compromising procedural safety.

## Materials and methods

Study design

This monocentric, two-arm, open-label, randomized crossover study was conducted under controlled laboratory conditions using two anatomically modified porcine bladder models. Each bladder was distended with either 180 mL of ambient air (GASCYS) or 180 mL of saline (LICYS). The order of cystoscopy (GASCYS vs. LICYS) was assigned via randomized group allocation to minimize order and learning effects.

Following each cystoscopy, participants completed a standardized questionnaire assessing endoscopic visibility on a five-point Likert scale (1=poor, 5=excellent). The questionnaire additionally assessed (i) perceived vision impairment attributable to hematuria (five-point Likert; 1=not impaired, 5=most impaired). The complete item wording and response anchors are provided in Appendix A After completing both cystoscopies, the participants answered another questionnaire assessing the ease of use (comparative rating versus the other modality). The complete item wording and response anchors are provided in Appendix B.

Participants with limited cystoscopy experience (≤10 prior procedures) received a brief, standardized five-minute training session covering scope handling, insertion technique, and common pitfalls.

Participants

Eligible participants were physicians employed in our institution who are either board-certified urologists or residents in either surgery or urology. Recruitment was conducted via an internal announcement. Written informed consent was obtained from all participants.

Randomization

Participants were block-randomized using a pre-generated randomization list, stratified by specialty: urologists (including both residents and board-certified urologists) and surgical residents. Within the urology group, further stratification was performed according to participants’ level of experience in cystoscopy. Allocation to intervention sequence occurred immediately prior to the procedure: in this cross-over design, Group A first performed GASCYS followed by LICYS, while Group B started with LICYS and then performed GASCYS.

Study setup

Two fresh porcine bladders with intact urethras were acquired from a local abattoir. The urethras were surgically shortened to approximately 10 cm to replicate human male anatomy and allow compatibility with standard clinical cystoscopes. To simulate macrohematuria, a blood-analog solution (28 mL Goodmark™ artificial blood (Goodmark Group, Kuurne, Belgium) mixed with 20 mL isotonic saline) was prepared. This commercially available artificial blood was selected to provide a stable, reproducible level of visual obscuration (color/opacity) across procedures, enabling standardized comparison of visibility between gas and liquid distension. As a non-biologic surrogate, it is not intended to reproduce the viscosity, coagulative behavior or full light scattering/absorption characteristics of native blood; thus, the model simulates optical impairment rather than the complete rheology of hematuria. From this blood-analog solution, 5 mL was injected into the bladder apex using a 20 mL syringe (Omnifix®, B. Braun, Melsungen, Germany).

**Figure 1:**
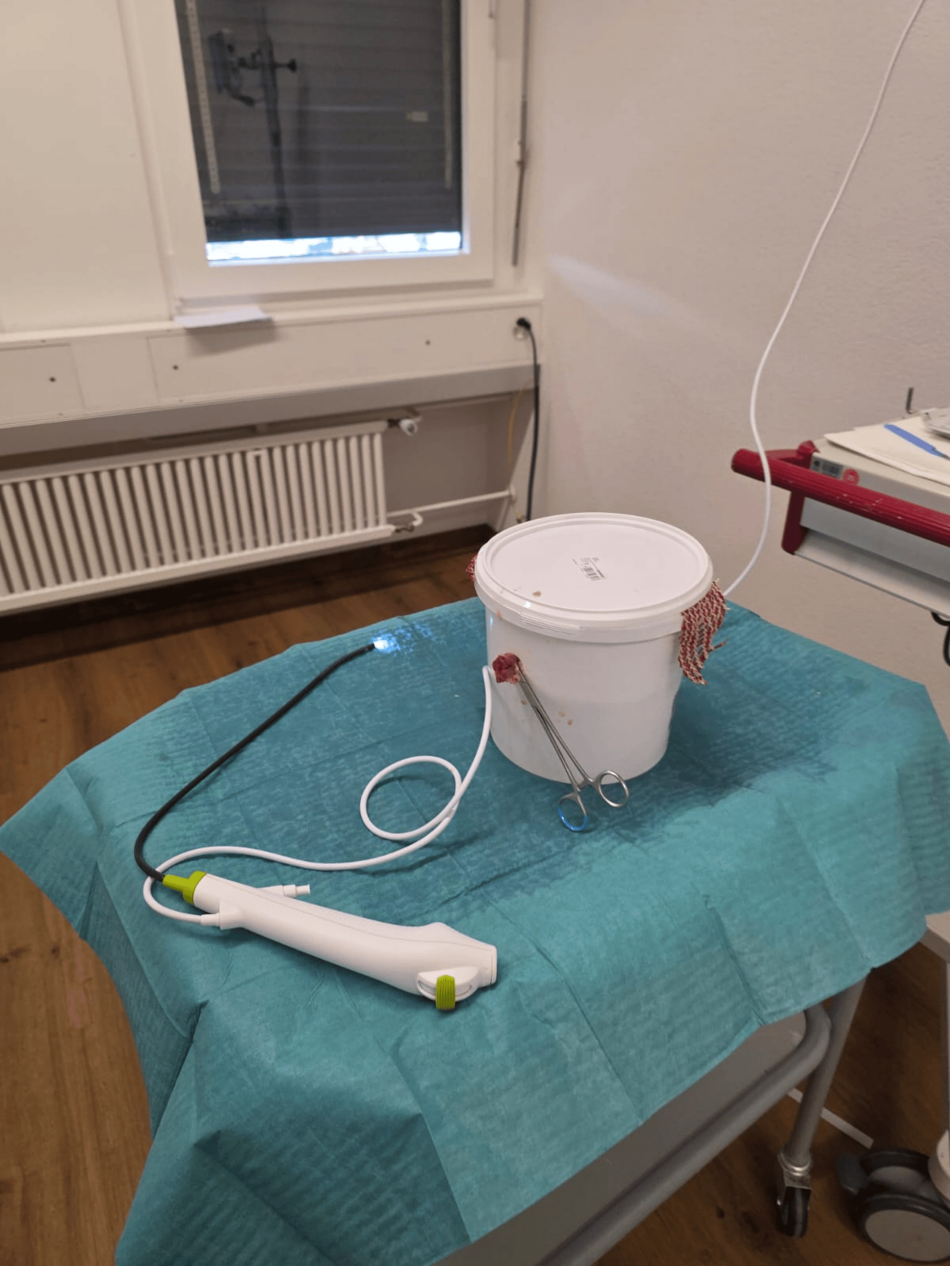
Experimental setup of simulated cystoscopy using a porcine bladder model A porcine bladder was suspended within a sealed 3.9-litre plastic container, with an artificial urethral access point positioned 3 cm below the rim. The bladder was filled with either 180 mL of ambient air or saline and injected with a blood-analogue solution (Goodmark™ (Goodmark Group, Kuurne, Belgium) mixed with isotonic saline) to simulate hematuria. Cystoscopy was performed using the Ambu® aScope™ 4 Cysto (Ambu GmbH, Bad Nauheim, Germany).

**Figure 2:**
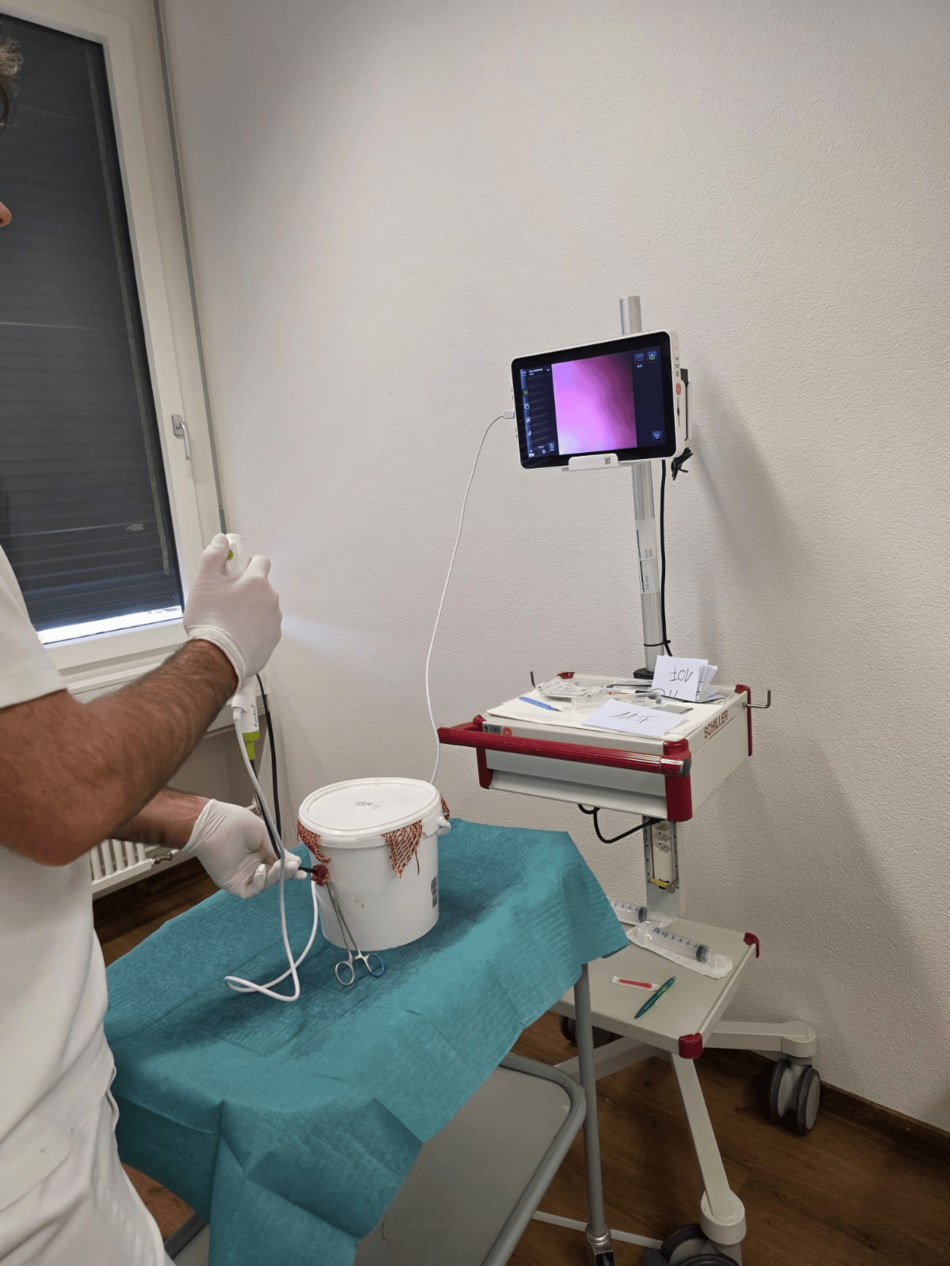
Execution of simulated cystoscopy using a porcine bladder model Figure shows a study participant conducting the procedure while visualizing the bladder interior on a connected monitor.

**Figure 3:**
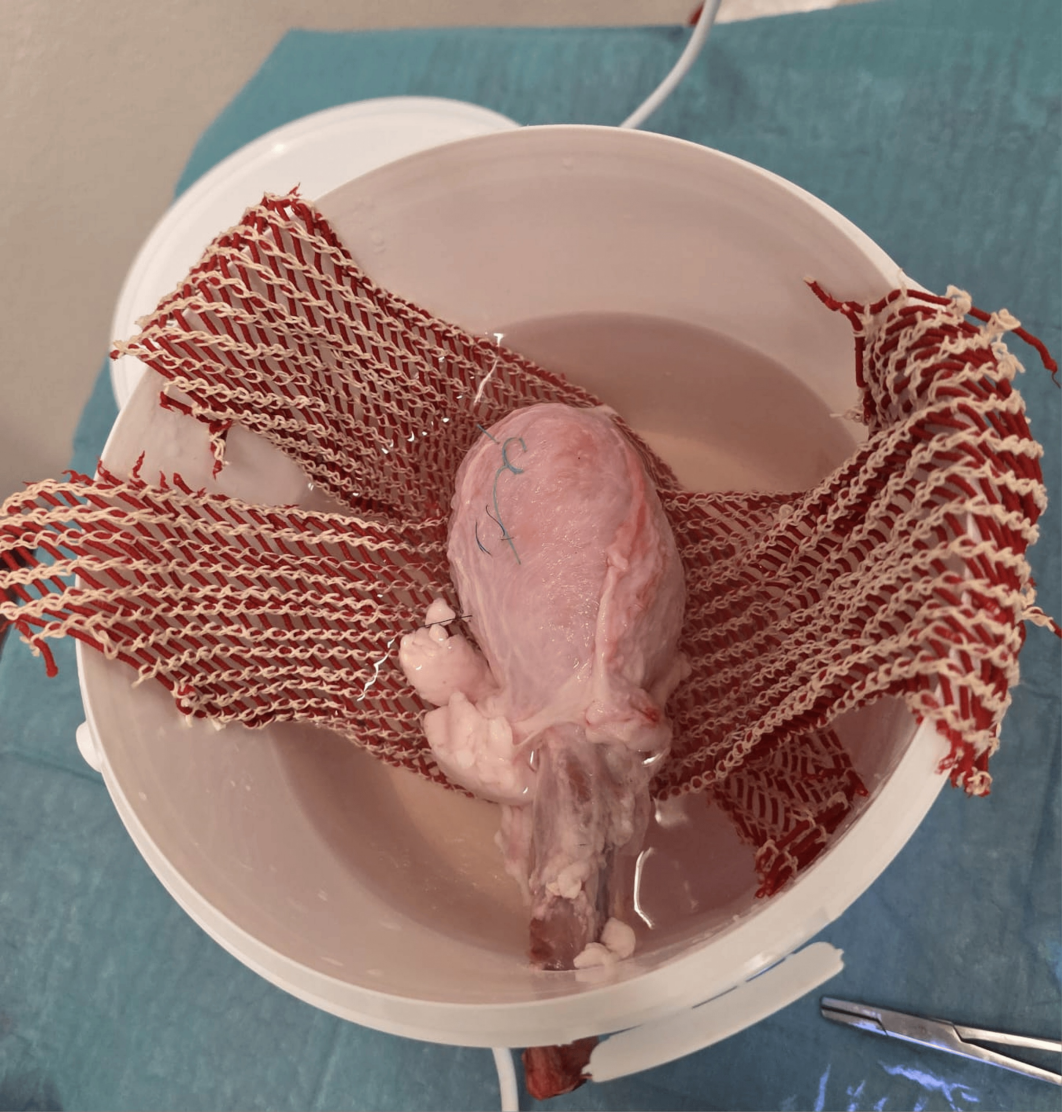
Preparation and mounting of the porcine bladder model for simulated cystoscopy Figure displays the mounted bladder in the experimental container, suspended with a fleece mesh to maintain anatomical positioning and ensure consistent endoscopic access during the procedure.

**Figure 4:**
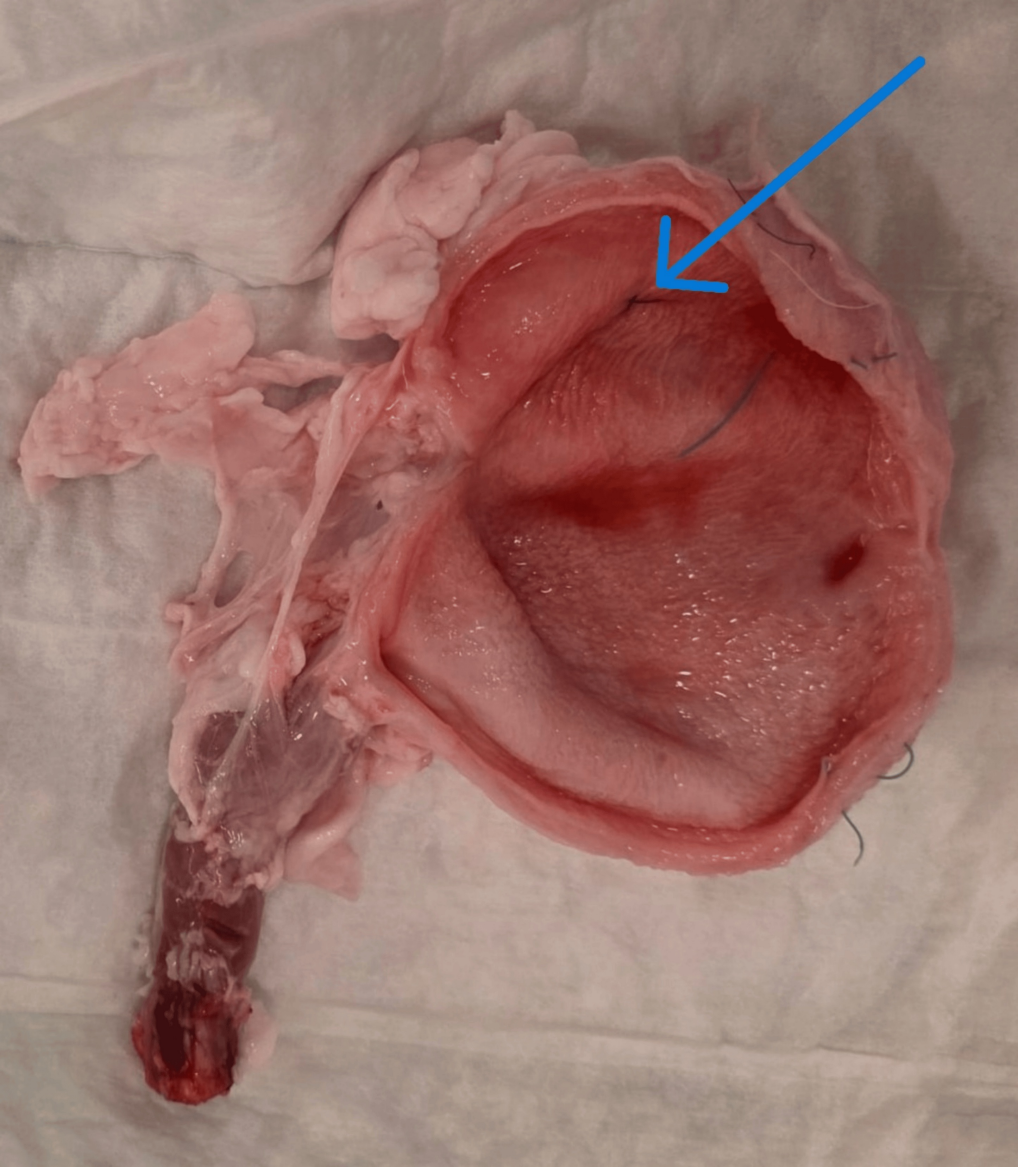
Preparation and mounting of the porcine bladder model for simulated cystoscopy Figure shows a freshly harvested porcine bladder with an intact, shortened urethra and one of the predefined intravesical markings (arrow) applied to the mucosal surface for visibility assessment.

Bladders were distended with either 180 mL of ambient air or saline using 60 mL syringes, intraluminal pressure was not directly measured. Each bladder contained three markings varying in color, texture, and diameter, placed in fixed locations to enable consistent visibility assessment. The setup was housed in 3.9 L plastic buckets (18.5 × 21 × 18 cm) with a 2 cm urethral access hole drilled 3 cm below the rim. The bladders were suspended using fleece mesh and secured with a needle holder to simulate realistic anatomical access. The bucket was filled with water and sealed with a lid to create a stable and enclosed environment (Figures 1-4).

Study procedure

Each participant performed both cystoscopy types (GASCYS and LICYS) on the porcine models. A single instructor (J.C.) inserted the cystoscope into the urethra and signaled the participant to begin. Time measurement commenced as the cystoscope entered the bladder and stopped either upon identification of all three markings (confirmed by the instructor) or after a 10-minute time limit.

Cystoscopies were performed using the Ambu® aScope™ 4 Cysto (Ambu GmbH, Bad Nauheim, Germany) and video-recorded for documentation and review. Subjective evaluations were collected immediately after each procedure using a paper-based questionnaire (see Appendices A and B) to minimize recall bias.

Outcomes and variables

The primary endpoint of the study was the time required to identify all three predefined intravesical markings. Secondary endpoints included assessments of subjective visibility, perceived visual impairment attributable to hematuria, and the incidence of iatrogenic trauma, as evaluated with a modified Objective Structured Assessment of Technical Skills (OSATS) checklist [19]. These study endpoints are summarized in Table 1.

**Table 1:** Study endpoints assessed OSATS: Objective Structured Assessment of Technical Skills. Adapted from Hu et al. [19].

Endpoint type	Description
Primary endpoint	Time (in seconds) required to identify all three markings
Secondary endpoints	Subjective visibility rating (Likert scale); Perceived visual impairment due to hematuria; Incidence of iatrogenic trauma (rated on a modified version of the OSATS checklist)

Statistical analysis

Analyses were conducted using RStudio (v2024.12.1+563, Posit Software, Boston, MA) on Windows (x64). All hypotheses were tested using two-tailed tests with a significance level of p<0.05 for the primary outcome. P-values for secondary outcomes were interpreted as descriptive and not adjusted for multiple comparisons. The primary outcome was analyzed using an independent samples t-test. Categorical secondary outcomes were compared between groups using Fisher's exact test. Ordinal visibility scores were analyzed using the Mann-Whitney U test.

## Results

Participant demographics

A total of 13 participants (100%) were enrolled and randomized: six (46.2%) to Group A (GASCYS first) and seven (53.8%) to Group B (LICYS first). The distribution of specialties was balanced, with Group A comprising three (50%) urologists (two residents (33.3%) and one board-certified (16.7%)) and three (50%) surgical residents, and Group B comprising four (57.1%) urologists (three residents (42.9%) and one board-certified (14.3%)) and three (42.9%) surgical residents (p=0.07). All surgical participants were residents.

The gender distribution was four (66.7%) males and two (33.3%) females in Group A, and two (28.6%) males and five (71.4%) females in Group B (p=0.29). Cystoscopy experience levels were comparable between groups: in Group A, three (50.0%) had no prior experience, one (16.7%) had performed at least 10 cystoscopies, and two (33.3%) had performed at least 100 cystoscopies; in Group B, four (57.1%) had no prior experience, zero (0.0%) had performed at least 10, and three (42.9%) had performed at least 100 procedures (p=1.0) These findings indicate no significant baseline differences between the groups (Table 2). 

**Table 2:** Baseline characteristics of participants in Group A and Group B This table presents demographic and professional characteristics of study participants stratified by randomization group. Variables include number of participants, residency programme (urology or surgery), sex, and level of cystoscopy experience. No statistically significant differences were observed between groups.

Parameter	Group A	Group B	P-Value
Number of Participants, n (%)	6 (46.2)	7 (53.8)	
Residency Programme – Urology, n	3	4	0.0669
Residency Programme – Surgery, n	3	3	
Sex – Male, n	4	2	0.2861
Sex – Female, n	2	5	
Experience – 0 prior procedures, n	3	4	1
Experience – ≥10 prior procedures, n	1	0	
Experience – ≥100 prior procedures, n	2	3	

Procedure duration

Mean procedure times were shorter for GASCYS (108.7 ± 147.0 seconds) than for LICYS (131.9 ± 135.9 seconds), although this difference did not reach statistical significance (p=0.68; 95% CI: -137.8 to 91.5 seconds). Procedure times were right-skewed, therefore we additionally report medians: GASCYS median (IQR) = 68 (39-106) seconds and LICYS median (IQR) = 104 (71-22) seconds (Table 3).

**Table 3:** Procedural duration for gas-based versus liquid-based cystoscopy Procedural time is reported in seconds (s) as median with interquartile range (IQR) and minimum–maximum values. GASCYS denotes the duration of gas-based cystoscopy, and LISCYS denotes the duration of liquid-based cystoscopy. No statistically significant difference in the duration has been detected.

Procedural times	GASCYS (s)	LYSCYS (s)
Minimum	11	44
Median (IQR)	68 (39-106)	104 (71-122)
Maximum	576	573

Procedure durations ranged from 11 to 576 seconds for GASCYS, and 44 to 573 seconds for LICYS. Importantly, all 13 participants (100%) successfully identified all three markings with both techniques, demonstrating that both methods allowed task completion under gross hematuria simulation.

Visibility and subjective difficulty

**Figure 5:**
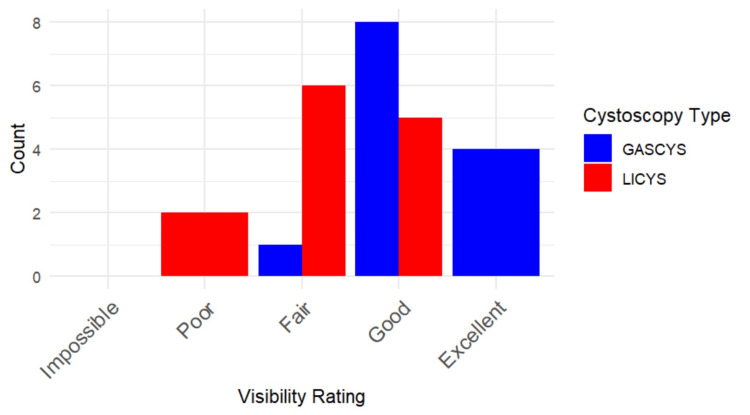
Subjective visibility ratings for gas- vs. liquid-based cystoscopy Bar chart illustrating participant-reported visibility scores on a 5-point Likert scale during gas-based cystoscopy (GASCYS, blue) and liquid-based cystoscopy (LICYS, red). GASCYS was more frequently rated as “good” or “excellent,” while LICYS received more “fair” and “poor” ratings.

**Figure 6:**
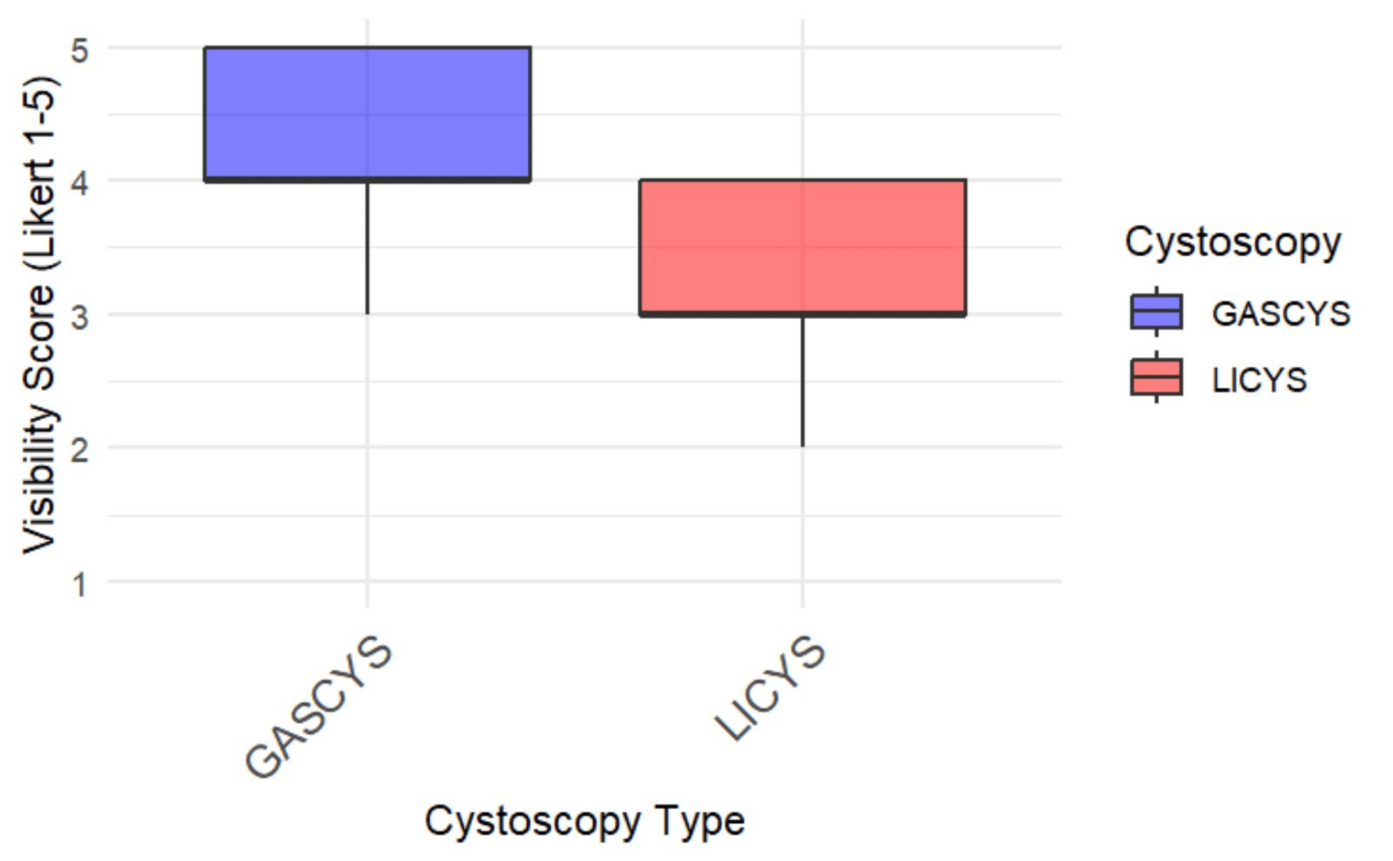
Box plot of visibility scores for gas- vs. liquid-based cystoscopy Box plot comparing subjective visibility ratings (Likert scale 1-5) between gas-based (GASCYS, blue) and liquid-based (LICYS, red) cystoscopy. GASCYS demonstrated higher median and overall visibility scores with less variability.

GASCYS was associated with significantly better subjective visibility scores than LICYS (p=0.0019; 95% CI: 0.000017, 2). Specifically, 92.3% of participants (12) rated visibility as "good" or "excellent" with gas, compared to only 38.5% (five) in the liquid condition. In contrast, 46.2% of the participants (six) in the LICYS group rated visibility as merely "fair" (Figures 5, 6).

**Figure 7:**
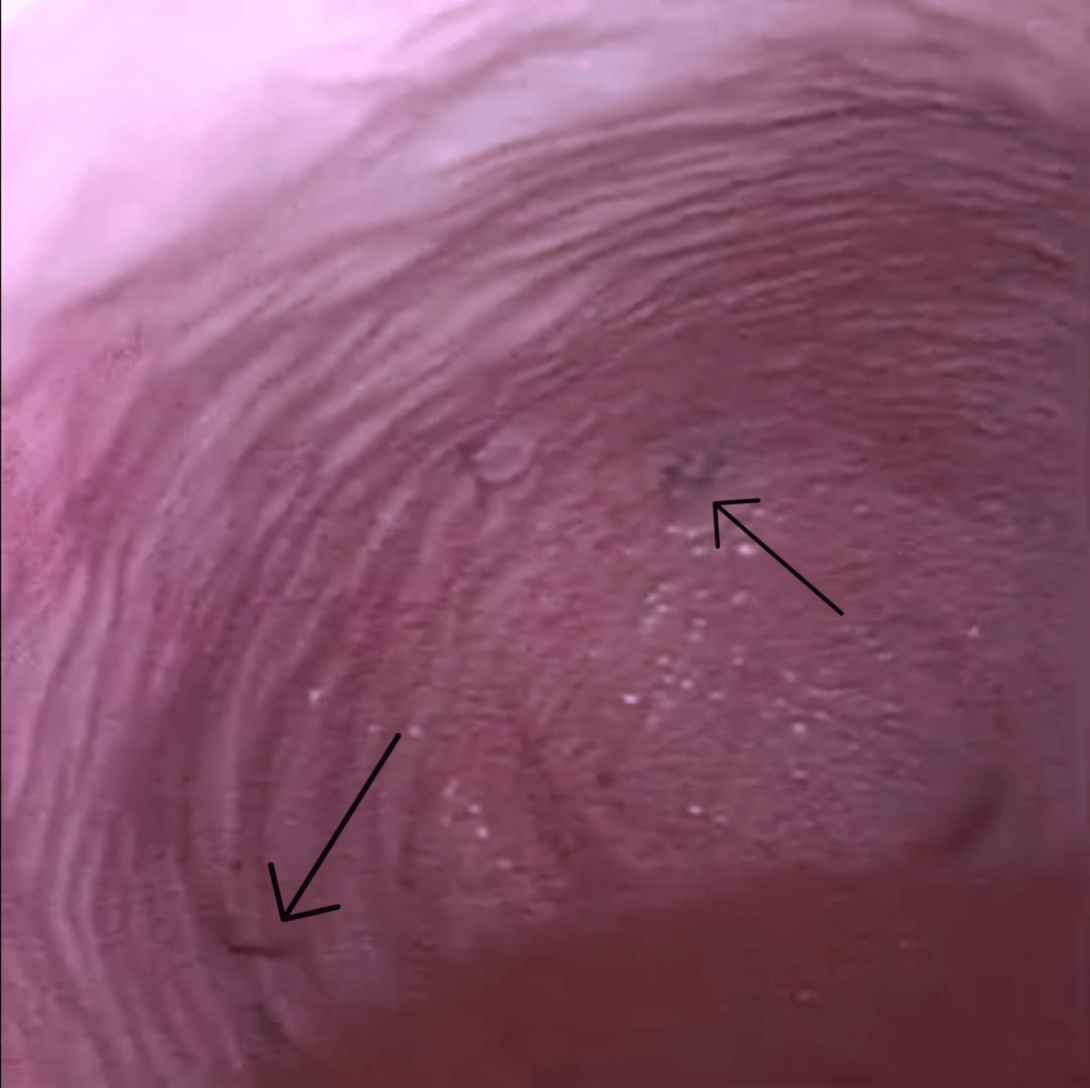
Endoscopic visualization of intravesical markings under gas-based cystoscopy Figure displays the endoscopic view during gas-based cystoscopy (GASCYS), showing two clearly visible predefined intravesical markings (arrows) against the mucosal surface with minimal visual distortion.

**Figure 8:**
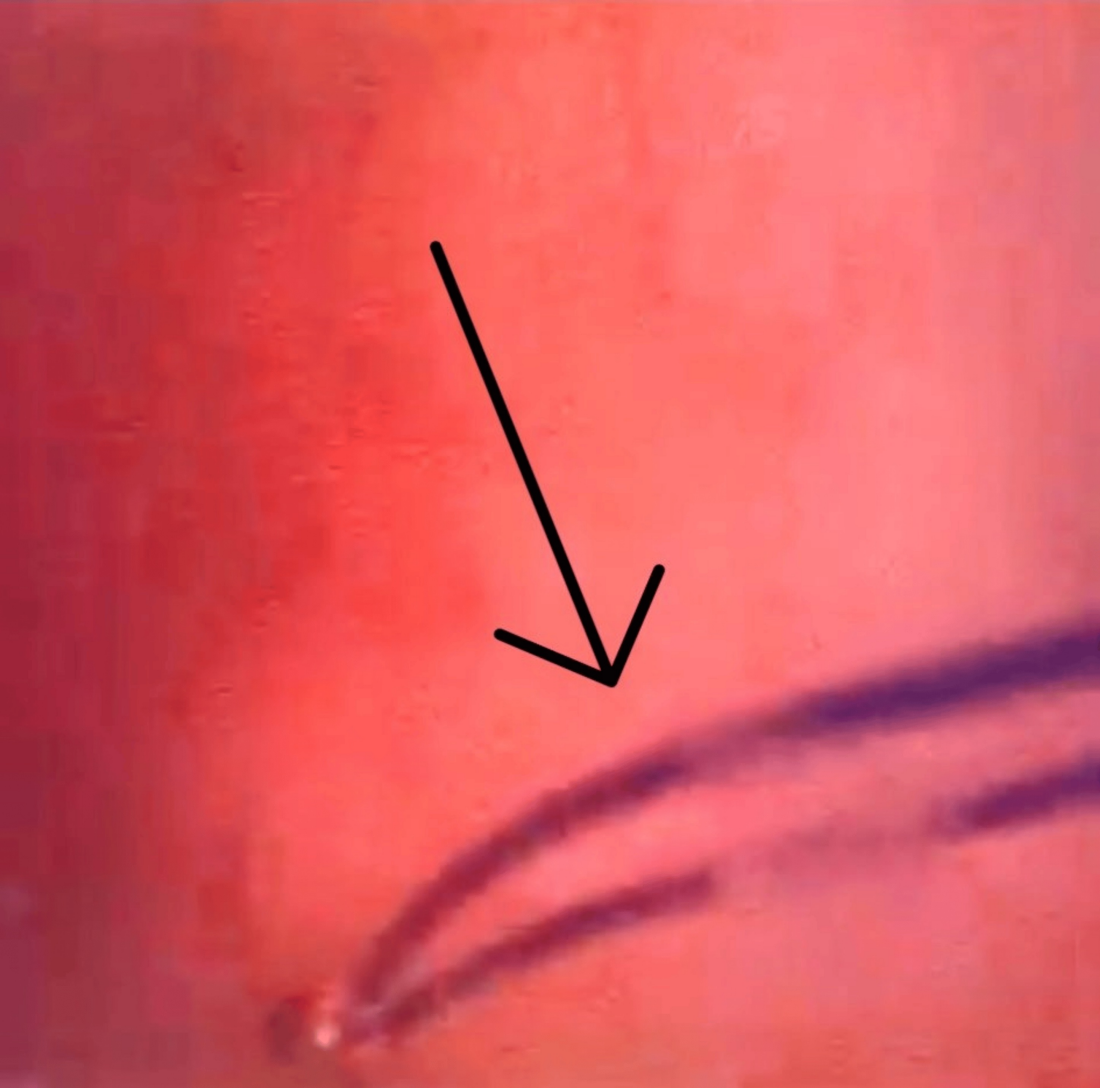
Endoscopic visualization of intravesical markings under liquid-based cystoscopy Figure illustrates the corresponding view during liquid-based cystoscopy (LICYS), where visibility of the same marking (arrow) is reduced due to turbidity and optical scattering from the simulated hematuria.

When directly comparing the two modalities, all 13 participants (100%) rated GASCYS (Figure 7) as easier to use than LICYS (Figure 8) (p=0.0012; 95% CI: 1.50, 2.00), indicating a clear subjective preference.

Of these, eight participants (61.5%) described GASCYS as “easier,” while five participants (38.5%) described it as “significantly easier” compared to LICYS.

Vision impairment

**Figure 9:**
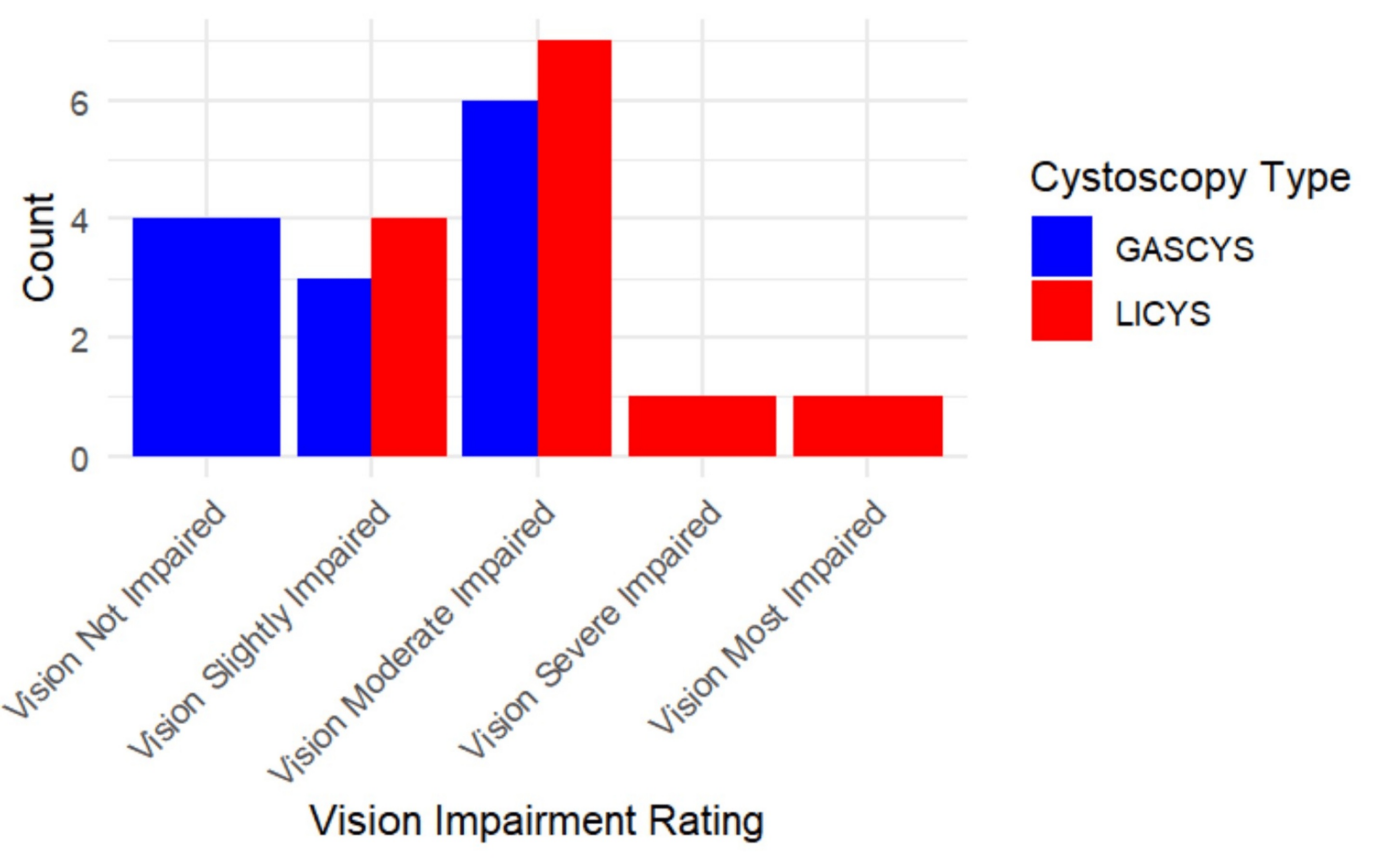
Subjective vision impairment ratings for gas- vs. liquid-based cystoscopy Bar chart showing participant-reported levels of vision impairment during cystoscopy (Likert-Scale, 1=not impaired to 5=most impaired). Gas-based cystoscopy (GASCYS; blue) was associated with fewer reports of severe impairment compared to liquid-based cystoscopy (LICYS; red), which had a higher frequency of moderate to severe visual disturbance.

**Figure 10:**
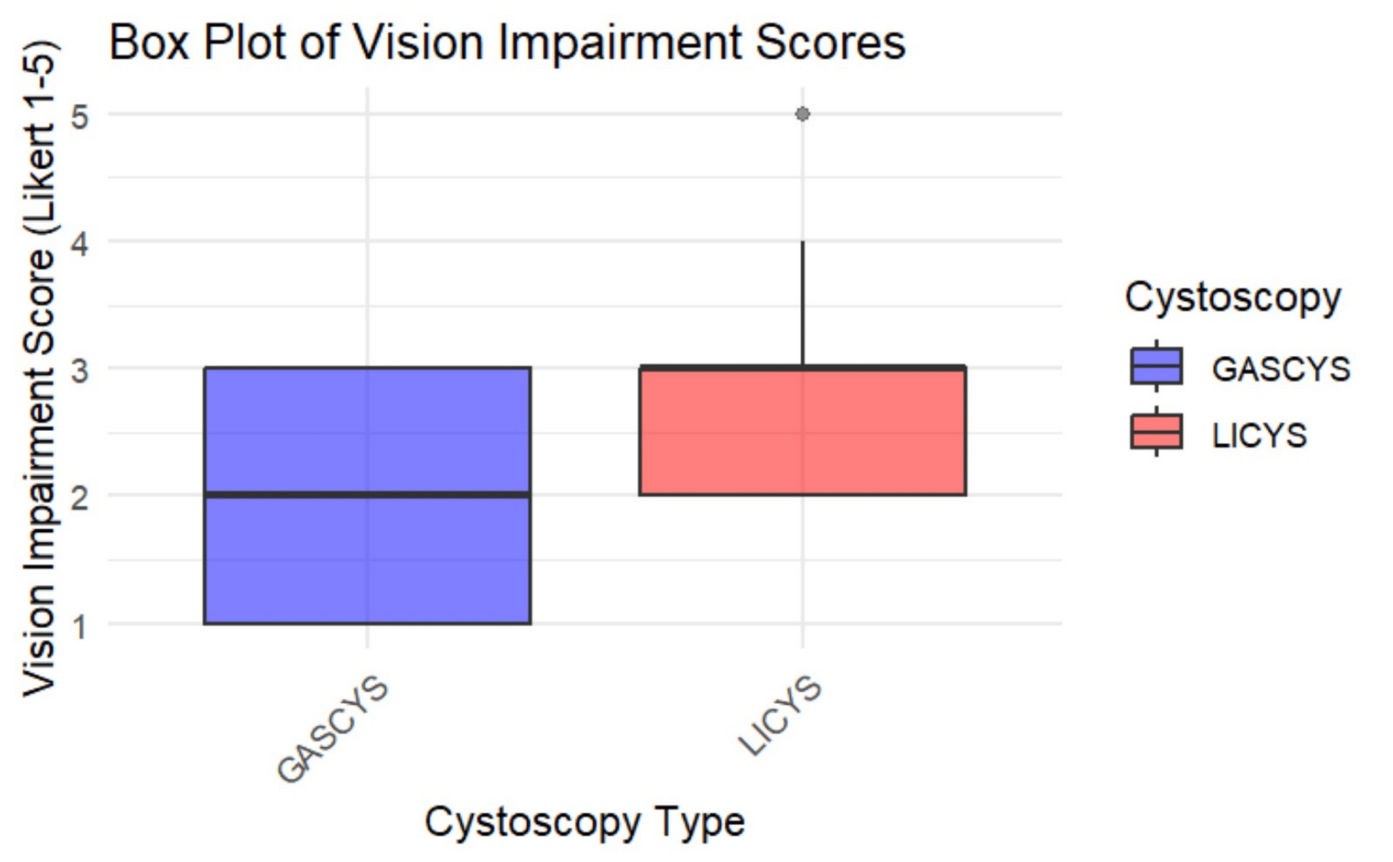
Box plot of vision impairment scores for gas- vs. liquid-based cystoscopy Box plot comparing subjective vision impairment ratings (Likert scale 1–5) between gas-based (GASCYS, blue) and liquid-based (LICYS, red) cystoscopy. GASCYS was associated with lower median impairment and reduced variability in scores.

Participants reported less visual impairment during GASCYS compared to LICYS, although this trend did not reach statistical significance (p=0.067; 95% CI: -2.00, 0.00004). In the gas condition, 30.8% of participants (four) reported no impairment, 23.1% (three) slight, and 46.2% (six) moderate impairment. In contrast, under the liquid condition, all participants reported some degree of visual impairment, with 53.8% of participants (seven) describing it as moderate and 15.4% (two) as severe. (Figures 9, 10)

## Discussion

In this pilot randomized crossover study, GASCYS demonstrated significantly improved subjective visibility and reduced procedural difficulty compared to conventional LICYS under simulated macrohematuria conditions. While the mean procedure duration was shorter with GASCYS, this difference did not reach statistical significance, likely due to the modest sample size. Nonetheless, the universal identification of all markings in both conditions confirmed the feasibility of GASCYS for diagnostic use.

The improved visibility during GASCYS is plausibly attributed to the fundamental physical properties of gas: unlike fluids, gas does not mix with blood [20], thereby preventing turbidity and preserving optical clarity. In contrast, LICYS is prone to blood admixture, impairing visibility, especially in cases of active or severe bleeding. These findings support using GASCYS as a potentially advantageous approach in patients with gross hematuria, where traditional fluid-based techniques may be compromised.

Although visualization was generally superior with gas, 38.5% of participants reported that residual fluid at the bottom of the bladder sometimes impaired mucosal assessment during GASCYS. In clinical settings, this can be exacerbated by the physiological urine production of the patient during cystoscopy. This limitation could be mitigated by aspirating residual fluid or adjusting patient positioning, such as tilting the examination table to improve fluid redistribution. Given the typical urine production of approximately 1.5-2.5 mL per minute in euvolemic adults [21], a single aspiration will often suffice to maintain a clear field over a short diagnostic examination (five to 10 minutes), although longer procedures, diuretics, or higher urine output may necessitate repeated aspiration.

Importantly, lesion behavior differs across media. Papillary or delicate small lesions may appear more voluminous in fluid but can partially collapse or adhere to the wall in gas, reducing visibility for very small tumors. This media effect has been quantified in a non-hematuric surveillance setting: air cystoscopy performed similarly to water cystoscopy at the patient level, correctly identifying all patients who required transurethral bladder resection; however, at the lesion level, air cystoscopy missed seven additional papillary implants, each smaller than 2 mm, compared with water [15]. These data indicate a numeric sensitivity gap for sub-2 mm implants in a non-hematuric context, plausibly because in water such superficial implants float and expand whereas in air they tend to adhere and appear flatter. Whether this lesion-level difference should be considered an “acceptable error margin” is context dependent: for lower-risk NMIBC follow-up, brief delays in detecting sub-2 mm implants may be acceptable if patient-level management is unaffected; for high-risk NMIBC surveillance, even sub-2 mm lesions can be clinically relevant, and a higher detection threshold is warranted [22]. Crucially, the cited difference pertains to non-hematuric follow-up conditions; under acute gross hematuria, media mixing and turbidity dominate visibility, and gas may regain a relative advantage by preventing admixture. Optical enhancement (e.g., narrow-band imaging) alongside gas could potentially mitigate the small-lesion sensitivity gap, a hypothesis requiring prospective testing in a clinical cohort.

Operational factors also merit consideration. Prior tamponade clearance and catheter placement can introduce superficial urothelial abrasions, creating confounders that may hinder lesion discrimination in both media. During ongoing hemorrhage, visibility typically deteriorates over time. Rigid cystoscopes allow rapid fluid exchange to restore clarity; flexible systems also permit irrigation/exchange, but generally at slower rates, which may blunt yet not negate the relative advantages of gas in persistent bleeding. Practical optical impairments differ by medium: lens fogging is uncommon with contemporary anti-fog protocols, whereas adherent water droplets, particularly during back-look maneuvers, are a more frequent fluid-related artifact and usually resolve with simple mechanical wiping against moist mucosa [23].

From a practical standpoint, the simplicity of GASCYS may facilitate rapid deployment in acute urologic settings. The bladder can be distended using standard 50-60 mL syringes filled with ambient air, requiring no specialized insufflation equipment. This approach could accelerate diagnostic workflows in patients with active macrohematuria, potentially reducing delays caused by prolonged irrigation or repeat procedures.

The risk of air or gas embolism is a theoretical concern with endoluminal insufflation. In clinical application, embolism is considered unlikely given the absence of direct insufflation near blood vessels and the long-standing use of air cystoscopy without recorded cases of embolic complications [13,14,24]. Should carbon dioxide (CO₂) be utilized as the distension medium, systemic CO₂ absorption may theoretically precipitate hypercapnia and resultant respiratory acidosis.

Intraperitoneal pneumoperitoneum and intravesical distension are anatomically and physiologically distinct: the peritoneum is a highly vascular serosal surface with substantial CO₂ uptake during laparoscopy [25] whereas the bladder lumen is lined by a high-resistance urothelial barrier and experimental data suggest low CO₂ permeability across the bladder [26]. Accordingly, clinically relevant CO₂ absorption during short diagnostic cystoscopy is expected to be limited, but future clinical studies should incorporate pressure-controlled insufflation and CO₂ monitoring to define safe operating parameters.

Our findings are consistent with those of Rana et al. [24], Ciudin et al. [15], and Oliveira et al. [14], all of whom reported improved visualization using gas. Notably, our crossover design minimized learning and sequence effects, strengthening the reliability of our comparisons. While Ciudin et al. [15] highlighted the potential of GASCYS, their study lacked standardization and was not comparative, as highlighted by Kumar et al. [18]. Oliveira et al. [14] demonstrated the safety of CO₂-based GASCYS using laparoscopic insufflation systems, achieving controlled intravesical pressure without adverse effects. Our results suggest that even ambient air, delivered manually, is sufficient for diagnostic distension. Oliveira et al. [14] further emphasized that existing laparoscopic insufflators can be adapted for GASCYS, eliminating the need for additional equipment. An aspect that may gain growing relevance, considering the rising healthcare expenditures [27]. Moreover, Rana et al. [24] and Ciudin et al. [15] suggested that gas-based techniques may be more cost-effective than fluid-based alternatives. This hypothesis requires further health economic evaluation in future studies. Beyond diagnostics, gas-based distension has shown promise in therapeutic procedures, such as bladder stone removal [17], suggesting a broader potential for clinical applications.

Strengths of this study include its randomized crossover design, use of standardized porcine bladder models, and real-time subjective evaluations, which minimized recall bias. However, the study has limitations: the small sample size reduces statistical power, and using an ex vivo porcine model may not fully capture the complexity of human cystoscopy under active bleeding conditions.

Blinding was not feasible due to the inherently different appearances of gas and fluid media. Moreover, participants were aware of the study’s aims, which may have influenced subjective assessments. Selection bias is also possible, as clinicians interested in endoscopic innovation may have been more likely to participate. Finally, despite block randomization, including participants with heterogeneous cystoscopy experience likely increased variability in procedure times; future studies may incorporate a run-in phase or restrict operators to a more homogeneous experience level when estimating performance differences.

Altogether, our data support a pragmatic, complementary approach in clinical practice: standard fluid-based cystoscopy remains first-line for irrigation, clot management, and volumizing small papillary lesions; GASCYS may serve as an adjunct when fluid-based visualization is non-conclusive or rapidly degrades under bleeding.

Following the successful implementation of this standardized experimental protocol, we plan to initiate a prospective clinical trial to assess GASCYS under real-world conditions. Additionally, the broader applicability of gas-based visualization techniques in endo-urologic interventions merits further exploration.

## Conclusions

This pilot ex vivo study proofs the concept of GASCYS by demonstrating that GASCYS is feasible in a standardized model and potentially advantageous for enhancing endoscopic visibility in simulated macrohematuria. All participants successfully completed the primary task of identifying predefined markings with both modalities. While the subjective benefits of GASCYS are compelling, the difference in procedure time did not reach statistical significance. These findings suggest that gas-based distension might serve as a complementary approach when fluid-based visualization is compromised.

The limitations of this study must be acknowledged when considering its implications. The ex vivo porcine model cannot fully replicate the complexity of human cystoscopy under active bleeding conditions, and blinding was not feasible due to the inherent differences between gas and fluid media. Further clinical evaluation is necessary to determine the effectiveness, safety parameters, and cost-effectiveness. Subsequent prospective trials in patient populations are required to validate the efficacy, safety profile, and potential applications of this technique before considering broader clinical implementation.

## Appendices

Appendix A: Questionnaire VIEW

**Table 4:** Likert-scale questionnaire items and response options This table lists the questionnaire items used to assess participant cystoscopy experience, visibility of the markings, degree of visual impairment, and the perceived difficulty of gas-based cystoscopy compared with conventional (liquid-based) cystoscopy. Responses were recorded on a 5-point Likert scale (1–5), with higher scores reflecting greater experience, better visibility, more visual impairment, as applicable to the item. The participant received the same questionnaire after each performed cystoscopy.

Rating Questions	1	2	3	4	5
How many cystoscopies has the participant already performed?	0	≥ 10	≥ 25	≥ 50	≥ 100
Visibility of the markings	Impossible	Poor	Fair	Good	Excellent
Degree of visual impairment during the procedure	Vision not impaired	Vision slightly impaired	Moderately impaired	Vision severely impaired	Vision most impaired

Appendix B: Perceived comparative difficulty

**Table 5:** Perceived difficulty of gas-based versus conventional (liquid-based) cystoscopy Participants rated the perceived difficulty of gas-based cystoscopy relative to conventional (liquid-based) cystoscopy using a 5-point Likert scale ranging from 1 (much easier) to 5 (much more difficult). Higher scores indicate greater perceived difficulty with gas-based cystoscopy compared with conventional cystoscopy. The participant received this questionnaire after having performed both cystoscopies.

	1	2	3	4	5
Gas-based cystoscopy compared with conventional (liquid-based) cystoscopy	Much easier	Easier	About the same	More difficult	Much more difficult
